# A comprehensive intervention following the clinical pathway of eating and swallowing disorder in the elderly with dementia: historically controlled study

**DOI:** 10.1186/s12877-017-0531-3

**Published:** 2017-07-14

**Authors:** Masahisa Arahata, Makoto Oura, Yuka Tomiyama, Naoe Morikawa, Hatsue Fujii, Shinji Minani, Yukihiro Shimizu

**Affiliations:** 1 Department of General Medicine, Nanto Municipal Hospital, 938 Inami Nanto, Toyama, 932-0211 Japan; 2 Department of Dentistry and Oral Surgery, Nanto Municipal Hospital, 938 Inami, Nanto, Toyama, 932-0211 Japan; 3 Department of Nursing-in-Ward, Nanto Municipal Hospital, 938 Inami, Nanto, Toyama, 932-0211 Japan; 4 Department of Community-based-Rehabilitation, Nanto Municipal Hospital, 938 Inami, Nanto, Toyama, 932-0211 Japan; 5 Department of Internal Medicine, Nanto Municipal Hospital, 938 Inami, Nanto, Toyama, 932-0211 Japan

**Keywords:** Comprehensive geriatric assessment, Decreased oral intake, Elderly, Dementia, Multidisciplinary team approach, Clinical pathway

## Abstract

**Background:**

Eating problems in patients with advanced dementia are strongly associated with their deteriorating survival. Food and drink intake in people with dementia may be supported by specific interventions, but the effectiveness of such interventions is backed by almost no evidence. However, comprehensive geriatric assessment (CGA) might potentially clarify the etiology of decreased oral intake in people with dementia; thus improving their clinical outcomes.

**Methods:**

This study was a single-arm, non-randomized trial that included historically controlled patients for comparison. We defined elderly patients with both severely decreased oral intake depending on artificial hydration and/or nutrition (AHN) and dementia as “Eating and Swallowing Disorder of the Elderly with Dementia (ESDED)”. In the intervention group, participants received CGA through the original clinical pathway with multidisciplinary interventions. This was followed by individualized therapeutic interventions according to assessment of the etiology of their eating problems.

**Results:**

During the intervention period (between 1st April 2013 and 31st March 2015), 102 cases of ESDED were enrolled in the study and 90 patients had completed receiving CGA. Conversely, 124 ESDED patient controls were selected from the same hospital enrolled during the historical period (between 1st April 2011 and 31st March 2012). Most participants in both groups were bedridden with severe cognitive impairment. For the intervention group, an average of 4.3 interventional strategies was recommended per participant after CGA. Serological tests, diagnostic imaging and other diagnostic examinations were much more frequently performed in the intervention group. Recovery rate from ESDED in the intervention group was significantly higher than that in the historical group (51% v.s. 34%, respectively, *P* = 0.02). The 1-year AHN-free survival in the intervention group was significantly higher than that in the historical group (28% v.s. 15%, respectively, *P* = 0.01). No significant difference between the two groups was found for 1-year overall survival (37% v.s. 28%, respectively, *P* = 0.08).

**Conclusions:**

Use of CGA with multidisciplinary interventions could improve the functional status of eating and allow elderly patients with severe eating problems and dementia to survive independently without the need for AHN.

**Trial registration:**

ISRCTN57646445, this trial was retrospectively registered on 8th December 2015.

**Electronic supplementary material:**

The online version of this article (doi:10.1186/s12877-017-0531-3) contains supplementary material, which is available to authorized users.

## Background

Eating problems in patients with advanced dementia are recognized as one of the greatest risk factors for their survival [1]. Eating problems including disturbed eating behavior, dysphagia, nausea, and anorexia, are associated with various pathological conditions such as dementia, stroke, sarcopenia, mental illness, and systemic diseases that disturb many physical functions [2–5]. Moreover, these pathological conditions have various underlying etiologies [6–8], all of which can cause decreased oral intake. However, the etiology of eating problems in each patient is often unclear because most patients with advanced dementia are facing the end-of-life and are usually recommended to receive only palliative or hospice care [9]. Decreased oral intake in patients with a disorder in the anticipatory phase may not only be associated with advanced dementia but also with reversible conditions such as anorexia caused by infectious diseases, acute organ failures, acute exacerbation of chronic diseases, as well as reversible dementia [10]. However, these patients’ eating abilities may improve, leading to better prognosis, and targeted interventions might reinstate them from facing the end-of-life. Keeping oral intake at the level of independence from artificial hydration and/or nutrition (AHN) might help maintain quality of life even at the end-of-life. Although AHN is not usually recommended for advanced dementia, it can assist with nutritional intake predominantly caused by a potentially reversible condition [11].

Meta-analysis showed almost no evidence for the effectiveness of specific interventions to directly support food and drink intake in people with dementia [12]. The single interventional approach applied for each study included in the meta-analysis might have been inadequate for improving oral intake [12] because the etiologies of decreased oral intake were heterogeneous and had not been properly assessed. Inadequate assessment of decreased oral intake may lead to inaccurately concluding that patients remain in an irreversible state, resulting in them receiving inappropriate AHN such as tube feedings. A national Japanese survey showed that approximately 200,000 dysphagia patients in geriatric long-term settings were dependent on tube feedings despite not having undergone adequate evaluation of swallowing function [13].

Comprehensive geriatric assessment (CGA) has been shown to be effective for improving functional status and prognosis in elderly patients [14–16]. In particular, CGA may clarify the etiologies of eating problems and provide individualized intervention with the potential to improve dementia patients’ decreased oral intake. Therefore, we developed a new medical care system based on CGA for severe eating problems in patients with dementia and investigated whether it could improve their clinical outcomes.

## Methods

### Design and setting

This study was a single-arm, interventional trial that included historically controlled patients for comparison. Participants hospitalized at Nanto Municipal Hospital between 1st April 2013 and 31st March 2015 (the intervention period) were enrolled into the intervention group. Hospitalized patients from the same hospital were retrospectively selected from between 1st April 2011 and 31st March 2012 (the historical period) and comprised the historical group. Historical group participants met all enrollment criteria and none of the exclusion criteria.

### Participants

Participants were required to meet all five of the following criteria for study inclusion; 1) hospitalized with dementia at age ≥ 70 years, 2) cognitive impairment (Mini–Mental State Examination [MMSE] <24, or Hasegawa Dementia Rating Scale - Revised [HDS-R] <21) [17, 18], 3) did not receive intravenous or surgical treatment in the past 7 days, 4) oral intake was ≤500 k-calories per day for ≥7 days, and 5) dependent on AHN for ≥7 days. Criteria 3) to 5) were assessed regardless of whether a participant was hospitalized. We defined patients with severely decreased oral intake as “Eating and Swallowing Disorder of the Elderly with Dementia (ESDED)”. Patients were excluded from participating in this trial if they met any of the following criteria; 1) already determined to depend on persistent tube-feeding, 2) presence of a morphological anomaly due to a tracheotomy or an operation to remove all or part of the larynx, and 3) had been receiving end-of-life care. These exclusion criteria were anticipated to disturb the outcomes of this study.

### Constitution of new medical care system and recruitment

**Fig. 1:**
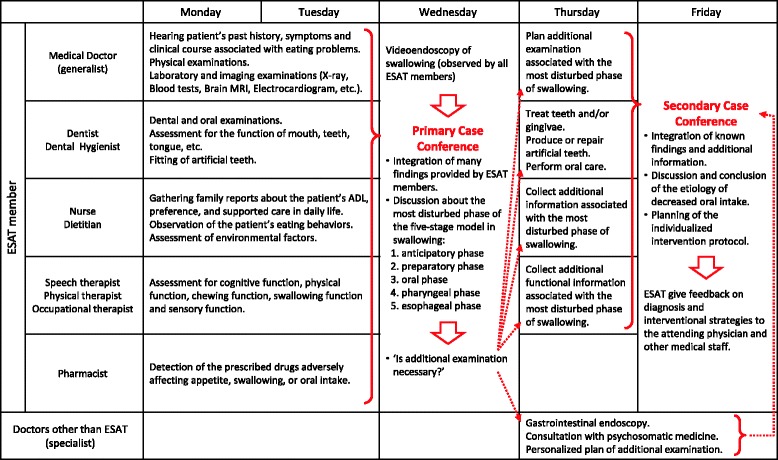
Overview of the clinical pathway for ESDED. During the clinical pathway process, the most disturbed phase of swallowing was identified and the etiology of the disturbance was discussed. Based on the diagnosis, the ESAT designed and informed of various interventional strategies to the attending medical staff. This clinical pathway was started on Monday because the examinations could occur only during the same working week. ADL, Activities of daily living; ESAT, Eating and swallowing assessment team; ESDED, Eating and swallowing disorder of the elderly with dementia; MRI, Magnetic resonance imaging

We organized a special multidisciplinary team for ESDED in April 2012, called the “Eating and Swallowing Assessment Team (ESAT)”, comprising medical doctors, dentists, dental hygienists, pharmacists, nurses (RN), speech therapists (ST), physical therapists (PT), occupational therapists (OT), dietitians, and certified care workers (CW). This multidisciplinary team developed a new system to examine various types of ESDEDs using the original clinical pathway (CP). This CP comprised integrated findings of assessments administered by each specialist, followed by diagnosis of the etiology of each ESDED case (Fig. 1). In the intervention period, the ESAT members selected candidates from all hospitalized patients by monitoring their oral intake and AHN. One of the ESAT members consulted with the attending physicians about recruiting potential participants into this study. With the attending physician’s permission, written informed consent was obtained from the participants. If participants suffered from advanced dementia, informed consent was obtained from their proxies.

### Intervention and follow up

Intervention group participants received CGA through the original CP with multidisciplinary interventions followed by individualized therapeutic interventions according to the etiological assessment of their eating problems. On the first to second day of the CP, the ESAT assessed each patient’s various functions (physical, cognitive, chewing, swallowing, and sensory), took their past medical and medication history, and obtained detailed information on their eating problems. The team performed physical and dental examination, urine and blood examination, chest X-ray, electrocardiogram, and magnetic resonance imaging of the brain (Fig. 1). In addition, the RN or ST observed each patient’s behavior and reaction during nursing at dinner and listed problems at each phase of the five-stage process model [19]. These comprehensive assessments with multidisciplinary interventions were filled in the prescribed form (Additional File 1: Fig. S1). On the third day of the CP, after videoendoscopic examination of swallowing, the ESAT held a conference to discuss the possible etiologies of eating problems in each patient based on the collected data. Thereafter, the ESAT planned further assessment or examination to identify the details of the suspected etiology. On the fourth to fifth day of the CP, the ESAT performed the planned assessment or examinations. The final diagnosis for the etiology of eating problems was made at a second conference held on the fifth evening of the CP. Based on the diagnosis, the ESAT proposed various intervention strategies to the staff providing medical care, nursing, and rehabilitation for the patients with ESDED. The ESAT observed whether the proposed strategies were actually carried out. The CW planned required daily home-based interventions to address eating problems for the patients, and introduced these plans to the patients’ caregivers before discharge. Clinical courses of the patients in the intervention group were observed prospectively by the ESAT for >1 year.

In the historically controlled group, patients had received usual medical care, in which attending physicians alone managed treatment and instructed other health professionals (such as the RN, PT, OT and ST) to provide required care and rehabilitations. The usual procedure of care for eating problems consisted of oral care, assistance when sitting and eating, and sometimes direct feeding. The usual rehabilitation program was composed of oropharyngeal, laryngeal, swallowing and speech training, mainly conducted by ST. The swallowing training consisted of indirect training (e.g., a range of motion exercises focused on the oropharyngeal and neck muscles, pseudo-supraglottic swallow, sham swallow, massaging the salivary gland, K-point stimulation, and the Shaker exercise) and direct training (feeding-swallowing foods and/or liquids at bedside). Therapists could provide one or more training regimens for each patient according to their own opinions. Additional techniques such as postural adjustments, chin-down/chin-tuck, cyclic ingestion, head rotation, diet modification, and use of capsaicin were adapted appropriately to each patient. The patients received these rehabilitations for at least 30 min a day. These patients’ clinical courses in the historical group were retrospectively followed without intervention according to this study’s protocol for up to 2 years after onset of ESDED.

### Outcomes

The hypothesis of this study was that comprehensive assessment with multidisciplinary interventions would contribute to improving the functional status of eating, leading to better survival of ESDED. The primary outcomes of this study were withdrawal from AHN and survival independent of AHN at 1 year after recovery from ESDED. Withdrawal from AHN was presented as a recovery rate (RR) in all eligible participants. Recovery was defined as a condition independent of AHN for ≥7 days. The decision to withdraw AHN was made by the attending physician in each case. Survival independent of AHN was presented as AHN-free survival (AHNFS). The endpoints for AHNFS were death by any cause and reversion to AHN dependence. The duration of AHNFS was measured between the day after AHN withdrawal and the day of achieving its endpoint. Duration of AHNFS in a patient who had failed to recover from ESDED was regarded as zero. The secondary outcome of this study was overall survival (OS) after onset of ESDED, which was measured between the day of onset of ESDED and the day of death by any cause. An additional outcome was RR in all cases of ESDED regardless of receiving interventions in each study period, which was analyzed retrospectively to verify the effect of the new medical care system with CGA and ESAT.

### Statistical analysis

In our previous study, 129 cases of ESDED had occurred during a 1-year observation period, and AHNFS was estimated to be approximately 10% at 1 year after recovery from ESDED; incidence of events was 90% per year. Because the incidence of events was intended to be reduced by 15% in the intervention group, 91 cases of ESDED in the intervention group would provide a power of 80% to show the superiority of our intervention at a two-sided alpha level of 0.05. We calculated that 2 years would be needed for this trial to enroll enough ESDED participants.

All numerical variables were tested for distribution normality using the Kolmogorov–Smirnov test, and data with *P*-values of <0.05 were regarded as non-normally distributed variables. Non-normally distributed variables were compared using the Mann–Whitney U test, while other variables were compared using t-tests between the intervention and historical groups. The proportions of categorical data in each group were compared using Fisher’s exact test or chi-square test as appropriate. Both AHNFS and OS were analyzed by survival assay using the Kaplan–Meier method, and were statistically compared between the two groups using Log-rank test. Logistic regression model was used to evaluate the influence of potential confounding factors on recovery from dependence on AHN. The factors were selected for multivariate analysis if the *P*-value in the univariate analysis was less than 0.20. These analyses were conducted according to the per-protocol principle. In addition, we retrospectively analyzed the RR of all ESDED cases in both the intervention and historical periods to assess the influence of the new diagnostic system and to exclude selection bias in the intervention period. This analysis was done according to the intention-to-treat principle. The odds ratio of RR was calculated to have improved in the intervention period. The retrospective dataset was obtained from a database, in which the ESAT had recorded the amount of oral intake and the procedure of AHN in all hospitalized patients.

All statistical analyses were performed using EZR (Saitama Medical Center, Jichi Medical University, Saitama, Japan, version 1.33), which is a graphical user interface for R (The R Foundation for Statistical Computing, Vienna, Austria, version 3.3.1) [20]. The EZR is a modified version of R commander (version 2.3–0), designed to add statistical functions frequently used in biostatistics. A two-sided *P*-value of <0.05 was considered statistically significant.

## Results

### Study population and participant characteristics

**Fig. 2:**
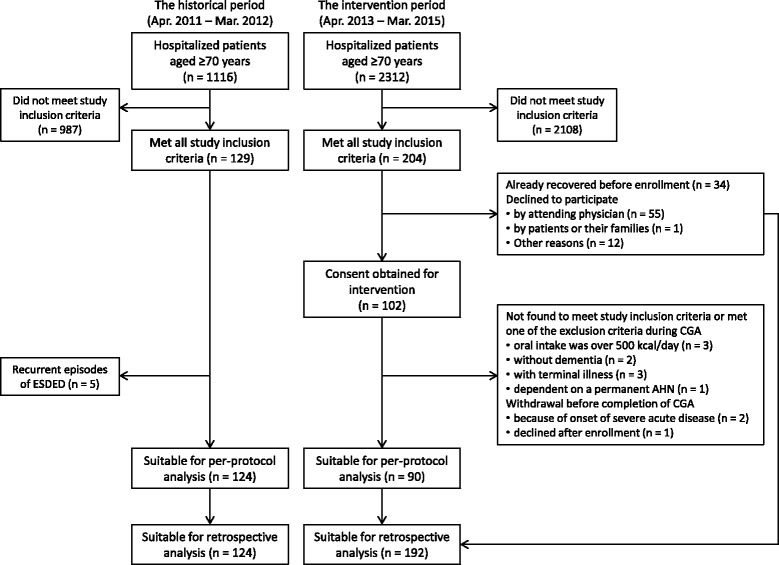
Enrollment of the participants. CGA, Comprehensive geriatric assessment; ESDED, Eating and swallowing disorder of the elderly with dementia

During the intervention period, approximately 9% of hospitalized patients aged ≥70 years met the study inclusion criteria. Up to 204 candidates remained after omitting patients who met study exclusion criteria, and 102 cases of ESDED consented to participate in this study. Twelve participants were withdrawn from the protocol because they either did not meet inclusion criteria or met one or more exclusion criteria during the CP. Therefore, 90 participants who had completed receiving CGA were eligible for analysis in the intervention group. Meanwhile, 129 cases of ESDED were found in the histological period and 124 of these patients were selected for study inclusion without duplication (Fig. 2).

Baseline characteristics of all patients are shown in Table 1. Age, sex, cognitive function and nutritional status evaluated by biochemical examination were not different between the two groups, but Barthel index (BI) was significantly higher in the intervention group (12 ± 21 v.s. 4.6 ± 13, *P* < 0.001). Most participants in both groups were bedridden with severe cognitive impairment. The proportion of acute diseases treated just before onset of ESDED was significantly different between the two groups (*P* = 0.005). In particular, the proportion of stroke in the historical group was slightly higher than that in the intervention group. By referring to their clinical course, these diseases were thought to cause the onset of ESDED. Some participants had malignancies as co-morbidity, but we estimated that most of them did not affect the onset of ESDED. A few patients had malignancies that affected and reduced oral intake, but these malignancies were exclusively at the advanced or incurable stage. Therefore, they were excluded from enrollment due to the exclusion criteria of “receiving end-of-life care”. Oral intake at baseline showed no difference between the two groups.

**Table 1:** Baseline characteristics of the participants

Characteristic	Intervention Group (*n* = 90)	Historical Group (*n* = 124)	*P*
Age	88.4 ± 6.9	88.2 ± 6.8	0.83
Male (n)	36	50	1.00
Female (n)	54	74	
Residence before admission (n)			0.45
Home	57	83	
Nursing facilities	32	37	
Other hospitals	1	4	
Diagnosis of dementia (n)			0.19
Alzheimer’s disease	25	33	
Vascular dementia	18	39	
Mixed dementia	25	20	
Frontotemporal dementia	1	3	
Lewy body disease	2	1	
Others or unknown	19	28	
Cognitive function			
HDS-R	3.3 ± 5.7	3.7 ± 6.2	0.77
MMSE	3.8 ± 6.2	4.4 ± 7.0	0.76
Daily life independence level of the elderly with dementia^a^ I or II (%)	27.8	28.2	1.00
Physical function			
Barthel Index	12 ± 21	4.6 ± 13	< 0.001
0 (n)	50	99	
5–15 (n)	19	13	
20–75 (n)	20	10	
80–100 (n)	1	1	
Not available (n)	0	1	
Daily life independence level (bedridden level) of the elderly with disability^a^ B or C (%)	98	99	0.57
Serological examinations			
Hemoglobin (g/dL)	10.9 ± 1.5	11.2 ± 2.1	0.15
Albumin (g/dL)	3.0 ± 0.5	3.0 ± 0.5	0.98
Diseases that affected the onset of ESDED			0.005
Infectious diseases (n)	44	68	
Stroke (n)	5	23	
Heart failure (n)	4	7	
Intestinal diseases (n)	3	5	
Absence (only dehydration and/or malnutrition) (n)	27	16	
Others (n)	7	5	
Oral intake (Kcal/day)	194 ± 160	168 ± 158	0.29
0 Kcal/day (n)	21	30	
1 to 100 Kcal/day (n)	12	25	
101 to 200 Kcal/day (n)	14	19	
201 to 300 Kcal/day (n)	15	20	
301 to 400 Kcal/day (n)	17	17	
401 to 500 Kcal/day (n)	11	13	

Data of numerical variables are presented as mean ± standard deviation

*ESDED* Eating and swallowing disorder in the elderly with dementia; *HDS*-*R* Hasegawa dementia rating scale - revised; *Kcal*/*day* kilo-calorie per day; *MMSE* Mini–mental state examination

^a^These scaling methods were defined by the Ministry of Health, Labour and Welfare in Japan [21]

### Interventions and outcome measures

The average number of the interventional strategies recommended by the ESAT was 4.3 per participant (Additional file 1: Fig. S2). In all intervention group participants, at least one of the interventional strategies recommended by the ESAT was carried out within 2 weeks. Serological tests, diagnostic imaging and the other diagnostic examinations were much more frequently performed in the intervention group than the historical group (data not shown). In particular, videoendoscopic examination of swallowing was performed more frequently in the intervention group than in the historical group (100% v.s. 20%, respectively, *P* < 0.001).

**Fig. 3:**
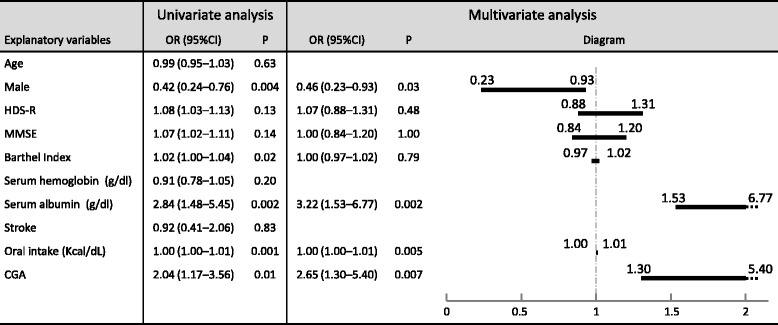
Odds ratio in independence from AHN (Logistic regression analysis). AHN, Artificial hydration and/or nutrition; CGA, Comprehensive geriatric assessment; CI, Confidential interval; HDS-R, Hasegawa dementia rating scale - revised; Kcal, kilo-calorie; MMSE, Mini–mental state examination; OR, Odds ratio

The outcome measures in this study are shown in Table 2. The RR of the intervention group was significantly higher than that of the historical group (51% v.s. 34%, respectively, *P* = 0.02). Logistic regression was used to evaluate the influence of potential confounding factors on withdrawal from ESDED, as shown in Fig. 3. Concentration of serum albumin at baseline was a large risk factor for independence from AHN (odds ratio 3.22, 95%CI 1.53 to 6.77, *P* = 0.002). Conversely, BI at baseline did not affect recovery, which was significantly different between the two groups.

**Table 2:** Clinical outcomes examined by the per-protocol analysis

Outcome	Intervention Group (*n* = 90)	Historical Group (*n* = 124)	*P*
AHN removal rate (recovery rate: RR) (%)	51	34	0.02
AHN-free survival rate (at 1 year) (%)^a^	28	15	0.01
Overall survival rate (at 1 year) (%)^a^	37	28	0.08
Final nutrition methods at discharge (n)			< 0.001
Eating without AHN	45	35	
Intravenous drip (peripheral or central venous)	41	63	
Tube feedings (PEG or nasogastric tube)	4	26	
Continuation of eating at discharge^b^ (%)	71	49	0.002
Duration of hospitalization (days)	79 ± 48	65 ± 57	0.003
Residence after discharge (n)			0.06
Home	21	46	
Nursing facilities	19	21	
Other hospitals	21	15	
Dead	29	42	

Data of numerical variables are presented as mean ± standard deviation

*AHN* Artificial hydration and/or nutrition; *PEG* Percutaneous endoscopic gastrostomy

**Fig. 4:**
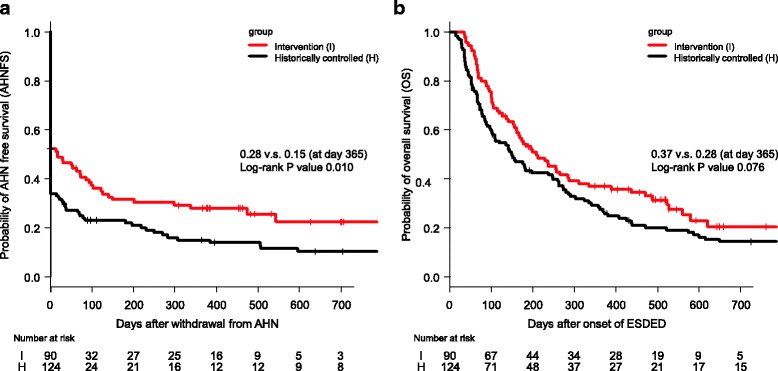
AHN-free survival and overall survival in patients with ESDED. Kaplan–Meier method was used for analysis of AHNFS (**a**) and OS (**b**). AHN, Artificial hydration and/or nutrition; AHNFS, AHN-free survival; ESDED, Eating and swallowing disorder of the elderly with dementia; OS, Overall survival

^a^Details of this measurement are described in Fig. 4

^b^If patients continued to eat at discharge, they were categorized as ‘continuation of eating’ regardless of dependence on AHN

The 1-year AHNFS in the intervention group was also significantly higher than that in the historical group (28% v.s. 15%, respectively, *P* = 0.01, Fig. 4a). No significant difference was found for 1-year OS between the two groups (37% v.s. 28%, *P* = 0.08, Fig. 4B). Retrospective analysis, in all ESDED cases during the historical and intervention periods, revealed that RR was significantly improved after the ESAT was established with our new care system including CGA (47% v.s. 34%, *P* = 0.02, Table 3).

**Table 3:** Clinical outcomes examined by retrospective analysis

Outcome	Intervention Group (*n* = 192)	Historical Group (*n* = 124)	*P*
AHN removal rate (recovery rate: RR) (%)	47	34	0.02
Final nutrition methods at discharge (n)			< 0.001
Eating without AHN	89	35	
Intravenous drip (peripheral or central venous)	87	63	
Tube feedings (PEG or nasogastric tube)	16	26	
Continuation of eating at discharge^a^ (%)	67	49	0.004

*AHN* Artificial hydration and/or nutrition; *PEG* Percutaneous endoscopic gastrostomy

^a^If patients continued to eat at discharge, they were categorized as ‘continuation of eating’ regardless of dependence on AHN

## Discussion

The present study showed that the CGA with a multidisciplinary team could determine the etiologies of ESDED in detail, which could help independence from AHN and improve AHNFS without reducing OS. This result indicates that focusing on the etiology of ESDED is important for improving clinical outcomes.

Many studies have verified the effectiveness of different interventions for oral intake in various care settings. However, Abdelhamid et al. reported finding no definitive evidence on the effectiveness, or lack of effectiveness, of specific interventions; furthermore, study samples were small and observation periods were short [12]. More patients with underlying conditions of acute or reversible diseases should have been included in previous studies. These reversible conditions in the elderly with eating problems and dementia are usually difficult to find because they typically cannot convey their symptoms. Therefore, the heterogeneity in etiologies of ESDED might have hampered the effect of specific simple intervention in previous studies. An assessment that estimates the causes or etiologies of ESDED can be considered essential to improving oral intake in ESDED. Especially when evaluating and managing dysphagia, a multidisciplinary approach is recommended; support for team approaches has already been reported [22–24]. Interventions such as postural adjustments, direct support of oral intake or modification of foods/liquids were not always regarded as a basic therapeutic treatment but were traditionally considered to be encompassed in symptomatic treatments or compensatory management [25]. Furthermore, it is difficult to make swallowing rehabilitation effective in patients with advanced dementia, such as those in our study populations, as shown by the low RR of the control group (Table 2). Conversely, assessment based on CGA could clarify the etiology of decreased oral intake, which helped us to individualize care and treatment. In addition, the interventions could become comprehensive with broad insights provided by a multidisciplinary team. Several advantages to multidisciplinary team interventions by refining health professionals’ roles and responsibilities within transition models in acute care settings have been reported, including: reducing emergency department re-admission rates, and reducing mortality and functional decline of older people [26]. As a result, our unique intervention method is more effective than specific interventions with a single procedure.

Although the methods for direct interventions for patients utilized in this study varied, the evaluation procedure for each patient (the CGA method) was uniformly performed by using CP. CPs are used broadly in medical care and are sometimes utilized as standardized protocols for optimizing and streamlining patient care [27]. Intervention using a CP was proven to be beneficial with a multidisciplinary team approach [28], and therefore we considered it was suitable for eating problems in patients with dementia. Additionally, the CGA method of this study is reproducible.

The BI at baseline, a physical function indicator, was significantly higher in the intervention group than in the historical group. Moreover, the proportion of those with stroke, an underlying disease that could affect the onset of ESDED, was significantly lower in the intervention groups (Table 1). However, neither BI nor stroke was a significant risk factor for independence from AHN according to logistic regression analysis (Fig. 3). Therefore, the higher BI and low frequency of stroke in the intervention group could not have contributed to the higher RR. Rather, sex, serum albumin concentrations at baseline, and the CGA strongly affected independence from AHN.

This study has some limitations. First, the factor that directly improved RR was unclear in each case. However, our new medical care system based on CGA has strong potential to improve the ability of eating and functional prognosis in patients with ESDED, and thus improve some survival measures (Fig. 4). Because all medical staff and caregivers in the two compared groups were almost identical with regard to their professional expertise and organizational structures, the new medical care system alone could be considered as having clear favorable outcomes for ESDED. Other outcome measures also support the effectiveness of our new medical care system (Table 3). These outcome measures in our study have not previously been reported in this field. We selected the primary and secondary outcome measures because the most valuable outcome measures for our study population were thought to be quality of life and prognosis. Although body weight is often selected as one of the index values concerning nutrition and outcome measures in patients with eating problems, the weight data could not be collected, especially in the control group because many of the patients were bedridden.

Second, the present study could not be carried out as a randomized controlled trial (RCT). Previous RCTs showed no evident effectiveness of a multidisciplinary team approach with CGA for elderly patients [16]. Considering the negative results from previous RCTs, we assumed that various medical care routinely provided by a trained multidisciplinary team in the control group might impair the strength of the interventions in the intervention group; thereby reducing the difference of outcome measures between the two groups in RCT. Furthermore, RCT could not be ethically approved for us to refrain from providing the best care or every possible method for treating ESDED in the control group because their prognosis was known to be poor. Therefore, we conducted this study as a historically controlled trial. Most studies evaluating the effect of a multidisciplinary team approach on dysphagia also treated retrospective datasets [22, 23, 29], pointing out that it was difficult to conduct a RCT using a multidisciplinary team approach [29].

Third, a selection bias may exist in this study because the attending physicians declined to enroll some participants (Fig. 2). The most common reason for refusal appeared to be that the physicians were concerned about their medical treatment being disturbed by ESAT or the study protocol. It is important to highlight that the physicians did not select patients for enrollment based on potential degree of benefits that could be obtained from this study. Therefore, it is unlikely that the physicians’ refusal to enroll some patients significantly affected the preferable results in the intervention group.

Fourthly, the decision to withdraw from AHN was dependent on each attending physician because we did not set a target value for intake. A universal target value of oral intake was difficult to set accurately for all subjects, despite established caloric targets being known to be associated with improved clinical outcomes [30–32]. Rather, we monitored survival rate to examine the validity of each withdrawal of AHN. If required AHN were inappropriately withdrawn, survival rate would reduce. In our study, however, overall survival was not shown to be significantly different between the two groups despite the intervention group achieving a higher RR from AHN (Fig. 4). Thus, AHNFS could not be analyzed precisely. However, we clearly demonstrated that CGA with multidisciplinary interventions contributed to improving the functional status of eating, resulting in a better independence rate from AHN. More studies with high evidence levels are required to conclusively determine the usefulness of comprehensive intervention for ESDED. We believe that higher quality assessments and interventions based on CGA will bring more favorable outcomes and reduce inadequate AHN for ESDED.

Ethical issues associated with AHN in elderly patients with advanced dementia are becoming hot topics in aging societies [33, 34], where physicians have been struggling with end-of-life care for these patients [35]. One embarrassing issue that remains to be solved is difficulty in judging reversibility of decreased oral intake in these patients, although their eating problems indicate poor prognosis [1]. Physicians may thus perform futile life-prolonging treatments with AHN or provide inappropriate end-of-life care without efficient interventions [13]. In addition, insufficient evidence exists regarding the outcome of AHN in older people with advanced dementia [36, 37]. However, we proved through this study that by performing the best assessment with the best procedure and that by providing appropriate care for each patient helped us to precisely judge reversibility. Furthermore, reversibility could improve the functional status of eating and help ESDED cases survive without the need for AHN. These two effects might assist in decision making for indications of AHN.

## Conclusions

A CGA with multidisciplinary interventions could improve the functional status of eating in elderly patients with severe eating problems and dementia to help them become independent from AHN. Therefore, we should not regard patients with ESDED as facing the end-of-life before adapting CGA for them.

## Additional file

Additional File 1:
**Fig. S1.** The prescribed CGA form. The prescribed form was completed with many findings from each ESAT professional within the initial 2 days. These information and data were shared among all ESAT members and proved useful for discussion to determine the etiology of ESDED. ACE, Angiotensin converting enzyme; BUN, Blood urea nitrogen; CTR, Cardio thoracic ratio; CRP, C-reactive protein; FAST, Functional assessment staging; HDS-R, Hasegawa dementia rating scale - revised; L-DOPA, L-3,4-dihydroxyphenylalanine; MMSE, Mini–mental state examination; MRI, Magnetic resonance imaging; MWST, Modified water swallowing test; NSAIDs, Non-steroidal anti-inflammatory drugs; OT, Occupational therapist; PPIs, Proton pump inhibitors; PT, Physical therapist; PVH, Periventricular hyperintensity; RSST, Repetitive saliva swallowing test; ST, Speech therapist; T-Cho, Total cholesterol; TSH, Thyroid stimulating hormone; WBC, White blood cells **Fig. S2.** Interventional strategies suggested by the ESAT. Interventional strategies were recommended in addition to already performed medical care, supportive care, and rehabilitation. The patterns of suggestions varied widely; only three patterns were plural (pattern number 42, 72, 78) and all others were singular. This means that the interventions based on this study were well individualized. The pattern number does not equate to the participants’ ID or sequence of enrollment in this study. ^a^ The description “medication for swallowing disorder” means both starting a medicine and stopping a medicine. The former was expected to positively affect swallowing, while the later was expected to disturb swallowing. ADL, Activities of daily living; AHN, Artificial hydration and/or nutrition; OT, Occupational therapist; PT, Physical therapist; ST, Speech therapist. (PPTX 66 kb)

## Abbreviations

AHN: Artificial hydration and/or nutrition
AHNFS: AHN-free survival
BI: Barthel index
CGA: Comprehensive geriatric assessment
CP: Clinical pathway
CW: Certified care worker
ESAT: Eating and swallowing assessment team
ESDED: Eating and swallowing disorder of the elderly with dementia
HDS-R: Hasegawa dementia rating scale – revised
MMSE: Mini–mental state examination
OS: Overall survival
OT: Occupational therapist
PT: Physical therapist
RCT: Randomized controlled trial
RN: Nurse
RR: Recovery rate
ST: Speech therapist
