# Supplementary material for: A comprehensive intervention following the clinical pathway of eating and swallowing disorder in the elderly with dementia: historically controlled study
Source: BMC Geriatr. 2017 Jul 14;17:146. doi: 10.1186/s12877-017-0531-3 (PMC5512987; doi:10.1186/s12877-017-0531-3)
Supplement: Additional File 1: — Fig. S1. The prescribed CGA form. The prescribed form was completed with many findings from each ESAT professional within the initial 2 days. These information and data were shared among all ESAT members and proved useful for discussion to determine the etiology of ESDED. ACE, Angiotensin converting enzyme; BUN, Blood urea nitrogen; CTR, Cardio thoracic ratio; CRP, C-reactive protein; FAST, Functional assessment staging; HDS-R, Hasegawa dementia rating scale - revised; L-DOPA, L-3,4-dihydroxyphenylalanine; MMSE, Mini–mental state examination; MRI, Magnetic resonance imaging; MWST, Modified water swallowing test; NSAIDs, Non-steroidal anti-inflammatory drugs; OT, Occupational therapist; PPIs, Proton pump inhibitors; PT, Physical therapist; PVH, Periventricular hyperintensity; RSST, Repetitive saliva swallowing test; ST, Speech therapist; T-Cho, Total cholesterol; TSH, Thyroid stimulating hormone; WBC, White blood cells Fig. S2. Interventional strategies suggested by the ESAT. Interventional strategies were recommended in addition to already performed medical care, supportive care, and rehabilitation. The patterns of suggestions varied widely; only three patterns were plural (pattern number 42, 72, 78) and all others were singular. This means that the interventions based on this study were well individualized. The pattern number does not equate to the participants’ ID or sequence of enrollment in this study. a The description “medication for swallowing disorder” means both starting a medicine and stopping a medicine. The former was expected to positively affect swallowing, while the later was expected to disturb swallowing. ADL, Activities of daily living; AHN, Artificial hydration and/or nutrition; OT, Occupational therapist; PT, Physical therapist; ST, Speech therapist. (PPTX 66 kb) [file 12877_2017_531_MOESM1_ESM.pptx]

## Slide 1
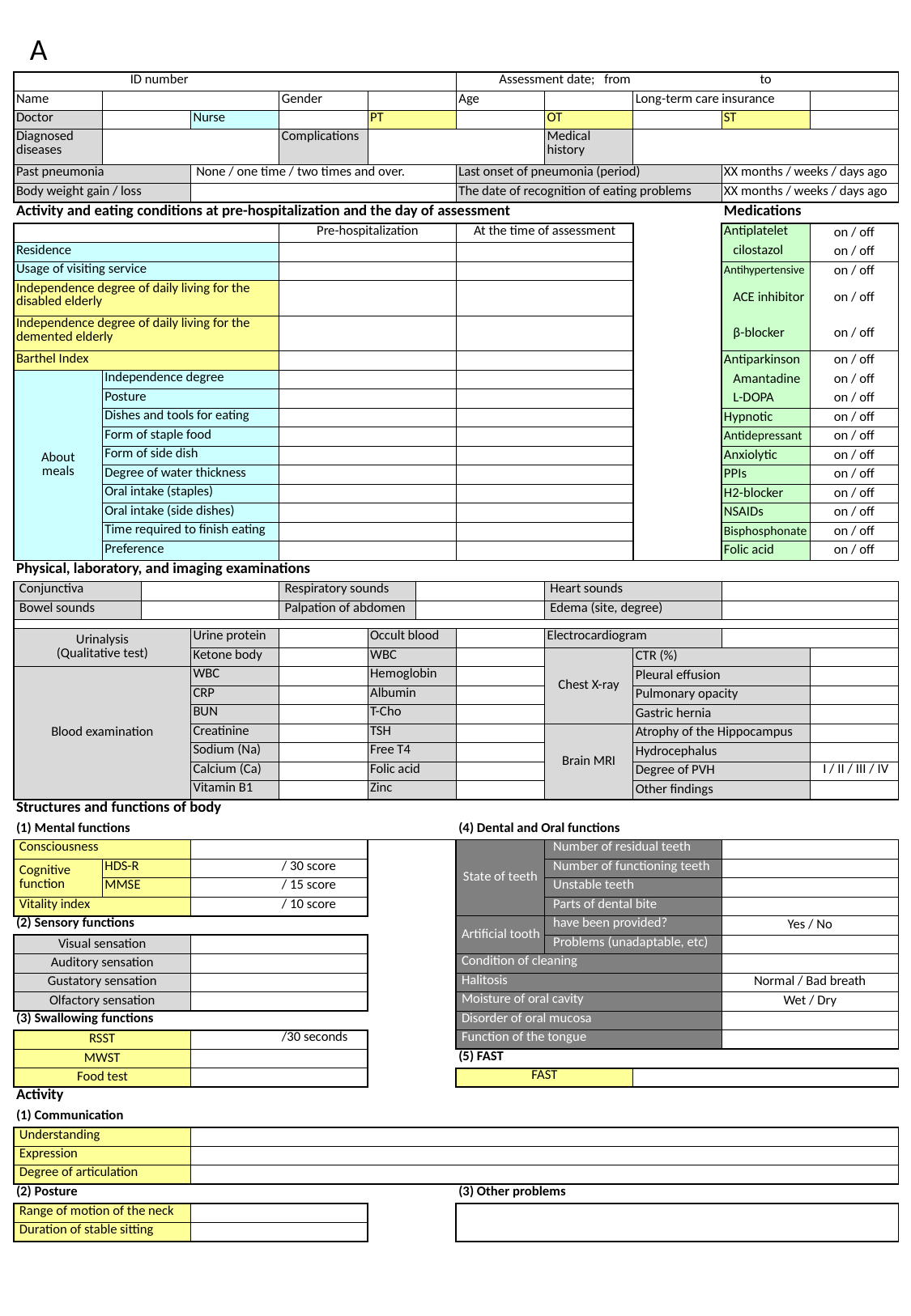

A
| ID number | | | | | | | Assessment date; from | | | to | |
| --- | --- | --- | --- | --- | --- | --- | --- | --- | --- | --- | --- |
| Name | | | | Gender | | | Age | | Long-term care insurance | | |
| Doctor | | | Nurse | | PT | | | OT | | ST | |
| Diagnosed diseases | | | | Complications | | | | Medical history | | | |
| Past pneumonia | | | None / one time / two times and over. | | | | Last onset of pneumonia (period) | | | XX months / weeks / days ago | |
| Body weight gain / loss | | | | | | | The date of recognition of eating problems | | | XX months / weeks / days ago | |
| Activity and eating conditions at pre-hospitalization and the day of assessment | | | | | | | | | | Medications | |
| | | | | Pre-hospitalization | | | At the time of assessment | | | Antiplatelet | on / off |
| Residence | | | | | | | | | | cilostazol | on / off |
| Usage of visiting service | | | | | | | | | | Antihypertensive | on / off |
| Independence degree of daily living for the disabled elderly | | | | | | | | | | ACE inhibitor | on / off |
| Independence degree of daily living for the demented elderly | | | | | | | | | | β-blocker | on / off |
| Barthel Index | | | | | | | | | | Antiparkinson | on / off |
| About meals | Independence degree | | | | | | | | | Amantadine | on / off |
| | Posture | | | | | | | | | L-DOPA | on / off |
| | Dishes and tools for eating | | | | | | | | | Hypnotic | on / off |
| | Form of staple food | | | | | | | | | Antidepressant | on / off |
| | Form of side dish | | | | | | | | | Anxiolytic | on / off |
| | Degree of water thickness | | | | | | | | | PPIs | on / off |
| | Oral intake (staples) | | | | | | | | | H2-blocker | on / off |
| | Oral intake (side dishes) | | | | | | | | | NSAIDs | on / off |
| | Time required to finish eating | | | | | | | | | Bisphosphonate | on / off |
| | Preference | | | | | | | | | Folic acid | on / off |
| Physical, laboratory, and imaging examinations | | | | | | | | | | | |
| Conjunctiva | | | | Respiratory sounds | | | | Heart sounds | | | |
| Bowel sounds | | | | Palpation of abdomen | | | | Edema (site, degree) | | | |
| | | | | | | | | | | | |
| Urinalysis (Qualitative test) | | | Urine protein | | Occult blood | | | Electrocardiogram | | | |
| | | | Ketone body | | WBC | | | Chest X-ray | CTR (%) | | |
| Blood examination | | | WBC | | Hemoglobin | | | | Pleural effusion | | |
| | | | CRP | | Albumin | | | | Pulmonary opacity | | |
| | | | BUN | | T-Cho | | | | Gastric hernia | | |
| | | | Creatinine | | TSH | | | Brain MRI | Atrophy of the Hippocampus | | |
| | | | Sodium (Na) | | Free T4 | | | | Hydrocephalus | | |
| | | | Calcium (Ca) | | Folic acid | | | | Degree of PVH | | I / II / III / IV |
| | | | Vitamin B1 | | Zinc | | | | Other findings | | |
| Structures and functions of body | | | | | | | | | | | |
| (1) Mental functions | | | | | | | (4) Dental and Oral functions | | | | |
| Consciousness | | | | | | | State of teeth | Number of residual teeth | | | |
| Cognitive function | HDS-R | | | / 30 score | | | | Number of functioning teeth | | | |
| | MMSE | | | / 15 score | | | | Unstable teeth | | | |
| Vitality index | | | | / 10 score | | | | Parts of dental bite | | | |
| (2) Sensory functions | | | | | | | Artificial tooth | have been provided? | | Yes / No | |
| Visual sensation | | | | | | | | Problems (unadaptable, etc) | | | |
| Auditory sensation | | | | | | | Condition of cleaning | | | | |
| Gustatory sensation | | | | | | | Halitosis | | | Normal / Bad breath | |
| Olfactory sensation | | | | | | | Moisture of oral cavity | | | Wet / Dry | |
| (3) Swallowing functions | | | | | | | Disorder of oral mucosa | | | | |
| RSST | | | | /30 seconds | | | Function of the tongue | | | | |
| MWST | | | | | | | (5) FAST | | | | |
| Food test | | | | | | | FAST | | | | |
| Activity | | | | | | | | | | | |
| (1) Communication | | | | | | | | | | | |
| Understanding | | | | | | | | | | | |
| Expression | | | | | | | | | | | |
| Degree of articulation | | | | | | | | | | | |
| (2) Posture | | | | | | | (3) Other problems | | | | |
| Range of motion of the neck | | | | | | | | | | | |
| Duration of stable sitting | | | | | | | | | | | |

## Slide 2
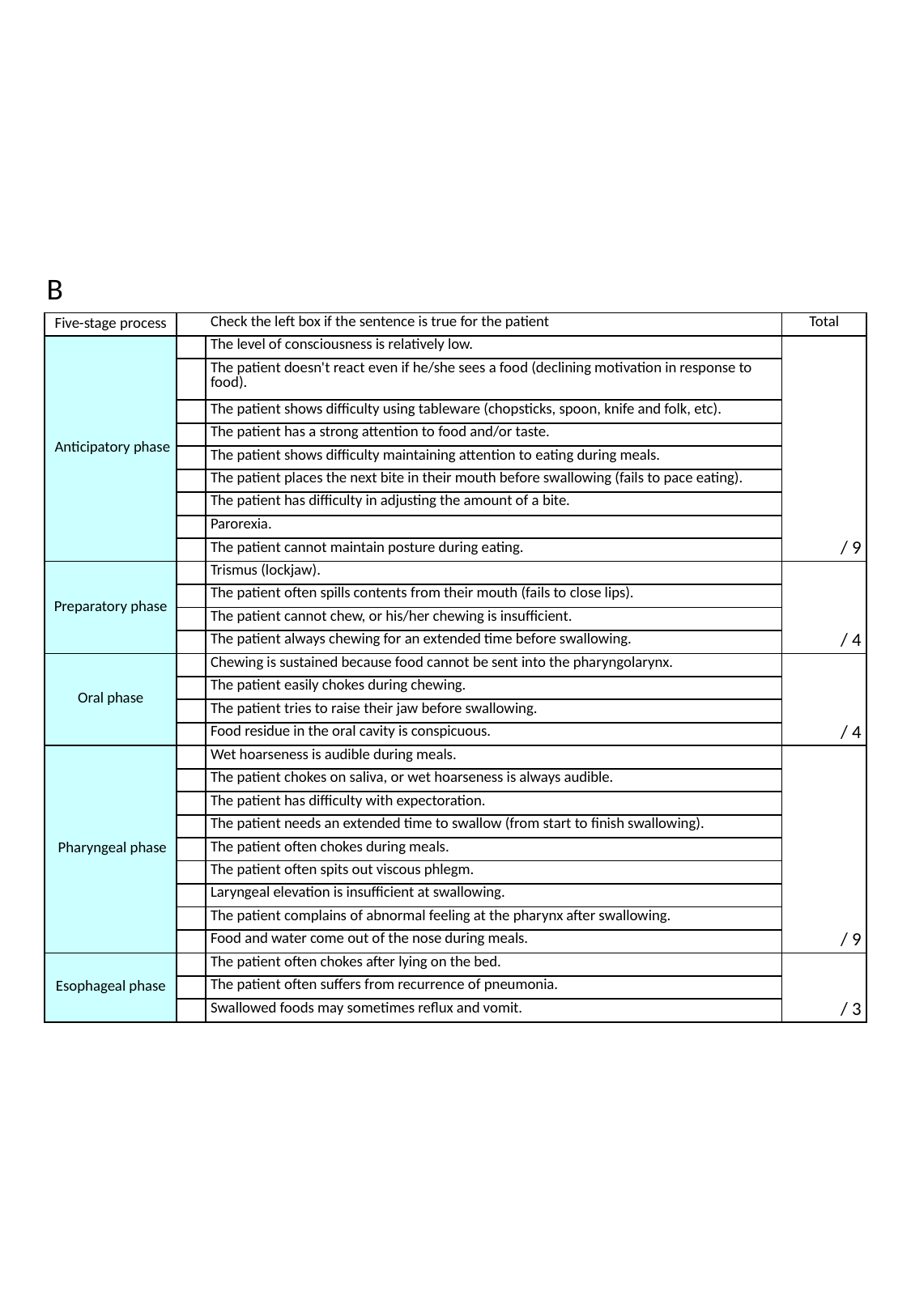

B
| Five-stage process | | Check the left box if the sentence is true for the patient | Total |
| --- | --- | --- | --- |
| Anticipatory phase | | The level of consciousness is relatively low. | / 9 |
| | | The patient doesn't react even if he/she sees a food (declining motivation in response to food). | |
| | | The patient shows difficulty using tableware (chopsticks, spoon, knife and folk, etc). | |
| | | The patient has a strong attention to food and/or taste. | |
| | | The patient shows difficulty maintaining attention to eating during meals. | |
| | | The patient places the next bite in their mouth before swallowing (fails to pace eating). | |
| | | The patient has difficulty in adjusting the amount of a bite. | |
| | | Parorexia. | |
| | | The patient cannot maintain posture during eating. | |
| Preparatory phase | | Trismus (lockjaw). | / 4 |
| | | The patient often spills contents from their mouth (fails to close lips). | |
| | | The patient cannot chew, or his/her chewing is insufficient. | |
| | | The patient always chewing for an extended time before swallowing. | |
| Oral phase | | Chewing is sustained because food cannot be sent into the pharyngolarynx. | / 4 |
| | | The patient easily chokes during chewing. | |
| | | The patient tries to raise their jaw before swallowing. | |
| | | Food residue in the oral cavity is conspicuous. | |
| Pharyngeal phase | | Wet hoarseness is audible during meals. | / 9 |
| | | The patient chokes on saliva, or wet hoarseness is always audible. | |
| | | The patient has difficulty with expectoration. | |
| | | The patient needs an extended time to swallow (from start to finish swallowing). | |
| | | The patient often chokes during meals. | |
| | | The patient often spits out viscous phlegm. | |
| | | Laryngeal elevation is insufficient at swallowing. | |
| | | The patient complains of abnormal feeling at the pharynx after swallowing. | |
| | | Food and water come out of the nose during meals. | |
| Esophageal phase | | The patient often chokes after lying on the bed. | / 3 |
| | | The patient often suffers from recurrence of pneumonia. | |
| | | Swallowed foods may sometimes reflux and vomit. | |
